# Cannabidiol-Loaded Lipid Nanoparticles Incorporated in Polyvinyl Alcohol and Sodium Alginate Hydrogel Scaffold for Enhancing Cell Migration and Accelerating Wound Healing

**DOI:** 10.3390/gels10120843

**Published:** 2024-12-20

**Authors:** Sarawut Lapmanee, Sakkarin Bhubhanil, Natthawut Charoenphon, Anjaree Inchan, Phichaporn Bunwatcharaphansakun, Mattaka Khongkow, Katawut Namdee

**Affiliations:** 1 Chulabhorn International College of Medicine, Thammasat University, Pathumthani 10120, Thailand; lapmanee@tu.ac.th; 2 Department of Basic Medical Sciences, Faculty of Medicine, Siam University, Bangkok 10160, Thailand; sakkarin.bhu@siam.edu; 3 Department of Anatomy, Faculty of Medical Science, Naresuan University, Phitsanulok 65000, Thailand; natthawutch@nu.ac.th; 4 Faculty of Medicine, Praboromarajchanok Institute, Ministry of Public Health, Nonthaburi 11000, Thailand; anjaree.in@gmail.com; 5 National Nanotechnology Centre, National Science and Technology Development Agency, Pathumthani 12120, Thailand; phichaporn@nanotec.or.th (P.B.); mattaka@nanotec.or.th (M.K.)

**Keywords:** cannabidiol, cell proliferation, injured wound, PVA/SA hydrogel

## Abstract

Chronic wounds represent a persistent clinical challenge due to prolonged inflammation and impaired tissue repair mechanisms. Cannabidiol (CBD), recognized for its anti-inflammatory and pro-healing properties, shows therapeutic promise in wound care. However, its delivery via lipid nanoparticles (LNPs) remains challenging due to CBD’s inherent instability and low bioavailability. This study developed and characterized a novel hydrogel scaffold composed of CBD-loaded LNPs (CBD/LNPs) integrated into a polyvinyl alcohol (PVA) and sodium alginate (SA) matrix, designed to enhance wound repair and mitigate inflammation. The characteristics of the hydrogel scaffold were observed including the degree of swelling and LNPs’ release profiles. Furthermore, in the results, CBD/LNPs displayed enhanced stability and reduced cytotoxicity compared to unencapsulated CBD. In vitro assays demonstrated that CBD/LNPs significantly promoted fibroblast migration in gap-closure wound models and reduced intracellular reactive oxygen species, supporting their potential as a biocompatible and efficacious agent for cellular repair and oxidative stress attenuation. In vivo experiments using adult male Wistar rats with aseptic cutaneous wounds revealed that treatment with CBD/LNP-PVA/SA hydrogel scaffold significantly accelerated wound closure relative to blank hydrogel controls, demonstrating a substantial reduction in the wound area over time. Histological analysis confirms notable improvements in skin morphology in wounds treated with CBD/LNP-PVA/SA hydrogel scaffold with evidence of accelerated epithelialization, enhanced collagen deposition, and increased dermal thickness and vascularization. Additionally, skin histology showed a more organized epidermal layer and reduced inflammatory cell infiltration in CBD/LNP-PVA/SA hydrogel scaffold-treated wounds, corresponding to a 35% increase in the wound closure rate by day 28 post-treatment. These findings suggest that CBD/LNP-PVA/SA hydrogel scaffolds facilitate inflammation resolution and structural wound healing through localized, sustained CBD delivery. The dual anti-inflammatory and wound-healing effects position CBD/LNP-PVA/SA hydrogel scaffold as a promising approach for chronic wound management. Future investigations are warranted to elucidate the mechanistic pathways by which CBD modulates the skin architecture and to explore its translational applications in clinical wound care.

## 1. Introduction

Chronic wounds constitute a major healthcare challenge worldwide, impacting millions and leading to increased morbidity and healthcare expenses [1,2]. Frequently associated with poorly managed conditions like diabetes and chronic inflammatory disorders, these wounds are prone to extended healing durations and an increased risk of complications [3]. Conventional wound management strategies often fall as being insufficient, necessitating innovative approaches derived from herbal and natural medicine to enhance wound healing outcomes [4].

Cannabidiol (CBD), a non-psychoactive compound derived from cannabis, has gained attention due to its anti-inflammatory, antioxidant, and wound-healing properties [5]. CBD has been shown to modulate the endocannabinoid system, potentially by binding to cannabinoid receptor type 1 and type 2, both of which are extensively expressed in the mammalian integumentary system, including skin and mucous membranes [6]. Disrupted endocannabinoid signaling is implicated in the pathogenesis of wound-related conditions such as dermatitis, psoriasis, and scleroderma; therefore, the therapeutic potential of CBD is addressing these complex skin disorders [7,8]. However, its therapeutic application is often limited by hydrophobicity, low bioavailability, and rapid degradation in physiological environments [9,10].

Recent advances in nanotechnology have facilitated innovative approaches to improve drug delivery systems for wound treatment. Various nanostructures, including liposomes, nanoparticles, nanofibers, and nanohydrogels, are being extensively studied to enhance the drug performance and optimize the drug encapsulation efficiency [11,12]. Among these, lipid nanoparticles (LNPs) have emerged as particularly promising carriers for bioactive compound delivery, valued for their biocompatibility, stability, and controlled release capabilities [13]. Therefore, encapsulating CBD within LNPs enhances its solubility, stability, and bioavailability, potentially improving its therapeutic efficacy [14,15]. To further innovate delivery for a wound healing approach, polyvinyl alcohol (PVA)/sodium alginate (SA) hydrogels can combine the beneficial properties of both polymers, creating a formulation ideal for wound dressing applications [16]. PVA provides robust mechanical strength, while SA enhances the physical and biological characteristics of the hydrogel [17,18]. This synergy enables a sustained, long-term release of encapsulated agents, as SA’s slow degradation rate prevents an initial burst release, making the CBD/LNP composite with PVA/SA hydrogel well-suited for therapeutic use.

This study aimed to evaluate the formulation of CBD/LNPs incorporating into PVA/SA hydrogels and their application in enhancing wound healing. The findings support the hypothesis that CBD/LNPs enhance the therapeutic efficacy when delivered via these hydrogels, demonstrating potential as an effective treatment for chronic wounds, characterized by improved biocompatibility, antioxidant and anti-inflammatory activity, and enhanced wound healing outcomes.

## 2. Results and Discusssion

### 2.1. Degree of Swelling of PVA/SA Hydrogel Scaffold

**Figure 1:**
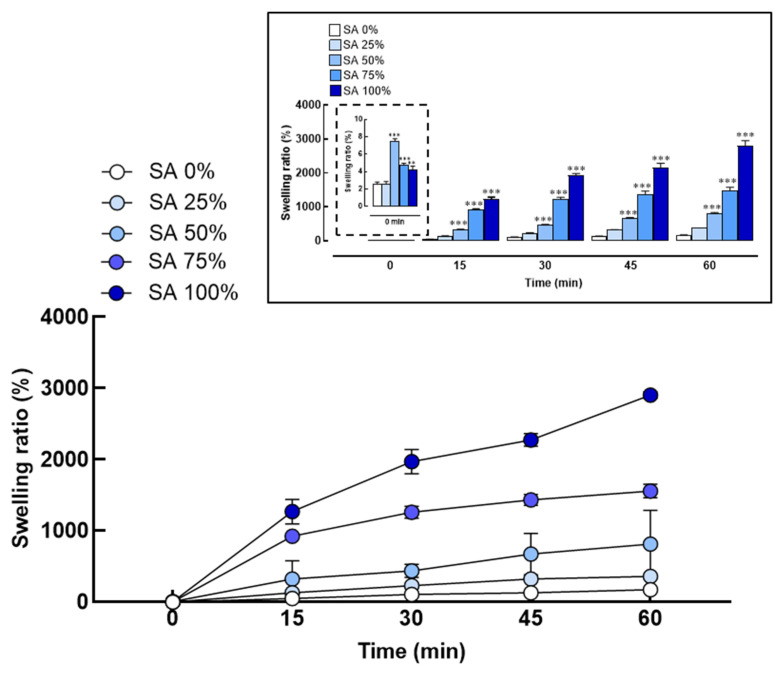
Swelling behavior of polyvinyl alcohol and sodium alginate (PVA/SA) hydrogel scaffold. Degree of swelling observed in hydrogel compositions containing varying percentages of sodium alginate (SA 25%, 50%, 75%, and 100%) and swelling ratio percentage measured at different time points (0, 15, 30, and 45 min). Significant differences were observed, with ** *p*  <  0.01 and *** *p*  <  0.001 compared to SA 0% at the starting point (0 min), as presented in the graph.

The swelling behavior of PVA/SA hydrogel scaffold was significantly influenced by the concentration of SA. As illustrated in Figure 1, a higher SA concentration resulted in greater swelling, enabling the hydrogel to absorb and retain more water. Higher hydrogel concentrations formed a denser polymer matrix, creating a network that facilitated increased water diffusion into the hydrogel.

These findings indicate that adjusting the hydrogel concentration can enhance the desired properties of the hydrogel scaffold. At 0% SA (PVA 100%), the response is minimal, with no significant increase over time. This lack of response indicates that 0% SA is ineffective at inducing any therapeutic effect, making it unsuitable for studies or applications that require measurable results.

At a 25% SA concentration, a moderate response is observed, with a clear, though modest, increase over time. Although the effect is weaker compared to higher concentrations, this concentration may be suitable for studies requiring a mild-to-moderate response without overwhelming stimulation.

The 50% SA concentration results in a more substantial increase in response over time, indicating a more pronounced effect. This concentration strikes a balance between the lower (25%) and higher (75%) concentrations, providing a robust therapeutic response without being excessively strong.

At the 75% SA concentration, the response becomes significantly more pronounced, especially after 30 min, with peak effects observed at 60 min. This concentration is suitable for applications requiring a strong and rapid effect, such as in acute inflammatory responses. However, the increased response at this concentration may also heighten the risk of overstimulation, toxicity, or unwanted side effects, particularly in sensitive systems. Therefore, precise dosing control is essential.

Finally, the 100% SA concentration produces the most substantial response, particularly at the later time points (45 and 60 min). While this concentration offers the strongest effect, it also carries the potential risk of oversaturation and toxicity, especially in more sensitive biological systems.

These results demonstrate a clear correlation between the SA concentration and the swelling behavior of hydrogels, with a higher SA content significantly enhancing the water absorption capacity of PVA/SA hydrogel scaffold. This finding highlights the pivotal role of SA in modulating the swelling properties of these hydrogels, which has important implications for the design and effectiveness of drug delivery systems. The swelling behavior is particularly critical for therapeutic efficacy, as it influences both the release kinetics of encapsulated CBD/LNPs and the hydrogel scaffold’s ability to maintain a hydrated environment at the wound site. This sustained swelling behavior supports the controlled release of the therapeutic agent, promoting cell migration and facilitating the wound healing process.

### 2.2. The Degree of Release of PVA/SA Hydrogel Scaffold

**Figure 2:**
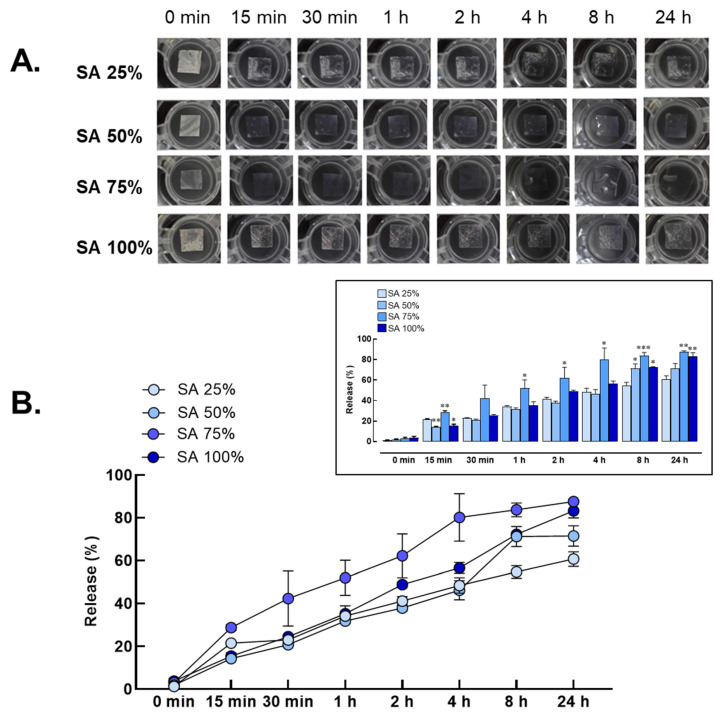
Release behavior of polyvinyl alcohol and sodium alginate (PVA/SA) hydrogel scaffold. (**A**) The degree of release was evaluated in hydrogel compositions containing varying percentages of sodium alginate (SA) incorporated with CBD/LNPs, including formulations with 25%, 50%, 75%, and 100% SA. (**B**) Release percentages measured at different time points (0 min, 15 min, 30 min, 1 h, 2 h, 4 h, 8 h, and 24 h). Significant differences were observed, with * *p* < 0.05, ** *p*  <  0.01, and *** *p*  <  0.001 compared to SA 25%, as presented in the graph.

The PVA/SA hydrogel scaffolds were evaluated for release using a freeze–thaw technique combined with CaCl_2_ to assess the impact on release kinetics over a 24 h period (Figure 2A,B). At the outset (0 min), all formulations exhibited no release (0.00%), establishing a baseline measurement. Among the varying concentrations of SA (25%, 50%, 75%, and 100%), the release profiles indicated that the 75% SA formulation achieved the highest release percentage, reaching approximately 88.46% after 24 h. Specifically, the 75% sodium alginate formulation demonstrated a significant release over time, with approximately 29.96% released at 15 min, 31.09% at 30 min, 47.03% at 1 h, 56.94% at 2 h, 68.40% at 4 h, 83.68% at 8 h, and ultimately, 88.46% at 24 h. These results suggest that the SA concentration significantly influences the release characteristics of PVA/SA hydrogel scaffold containing CBD/LNPs, with higher concentrations leading to increased release percentages. Additionally, the combination of freeze–thaw techniques and CaCl_2_ enhanced the hydrophilic properties of the hydrogels, improving water absorption and facilitating the release of encapsulated CBD/LNPs.

### 2.3. Formulation of CBD/LNPs

**Figure 3:**
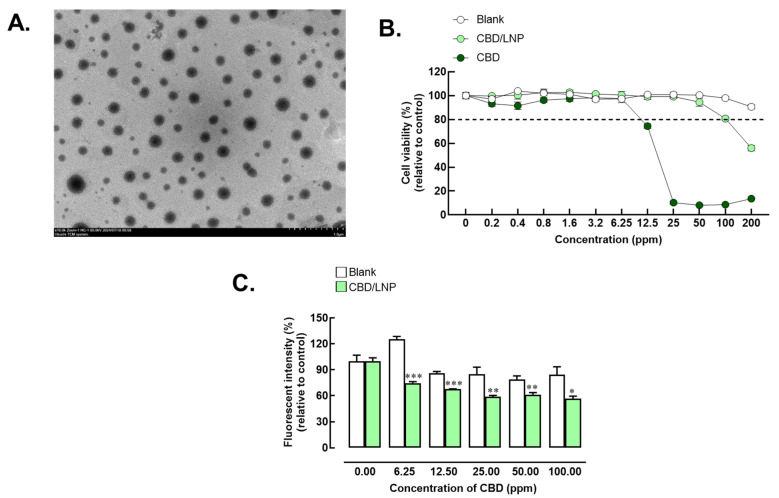
Characteristics, cytotoxicity, and antioxidant activity of cannabidiol-loaded lipid nanoparticles (CBD/LNPs). (**A**) Morphology of CBD/LNPs as demonstrated by Transmission Electron Microscopy (TEM); (**B**) Cell viability relative to the control in fibroblast-cultured cells exposed to CBD/LNPs; (**C**) Radical oxidative stress levels induced by hydrogen peroxide exposure were measured by the fluorescent intensity of dichlorofluorescein (DCF) relative to the control group, referring to untreated cells exposed only to the medium without any treatment. The analysis was conducted in fibroblast cultures treated with CBD/LNPs. Data are expressed as mean  ±  SEM (*n* = 4). * *p*  <  0.05, ** *p*  <  0.01, and *** *p*  <  0.001 compared to the blank group.

The morphological characteristics of CBD/LNPs were spherical in shape and exhibited homogeneity, as observed in the transmission electron microscopy images (Figure 3A). The stability of CBD/LNPs was evaluated over a 2-month period at three different temperatures: 4 °C, 25 °C, and 40 °C (Table 1). After 1 month, the average particle sizes measured were 131.9 nm, 136.8 nm, and 132.5 nm, respectively, with no significant changes observed compared to the initial measurements. The PDI remained stable at 0.161, 0.170, and 0.158 across the three temperatures, indicating a narrow size distribution. The zeta potential values at 4 °C, 25 °C, and 40 °C were −34.8 mV, −31.8 mV, and −31.4 mV, respectively, suggesting good stability of the nanoparticle dispersion. After 2 months, the average particle sizes increased slightly to 135.7 nm, 136.7 nm, and 134.2 nm at the corresponding temperatures, with these changes being statistically insignificant. The PDI values increased to 0.182, 0.192, and 0.158, still reflecting a relatively narrow size distribution. The zeta potential remained stable at −32.0 mV, −30.9 mV, and −27.3 mV, indicating that the nanoparticles maintained their electrostatic stability throughout the study. Therefore, CBD/LNPs demonstrated good stability over a 2-month period, with minimal changes in the particle size, PDI, and zeta potential. Despite a slight decrease in the encapsulation efficiency, these findings support the potential use of CBD/LNPs encapsulated in formulated hydrogels for therapeutic applications, ensuring efficacy and stability over time.

### 2.4. Cytotoxicity of CBD/LNPs

The cytotoxicity of CBD/LNPs incorporated into hydrogels was systematically evaluated in dermal fibroblast cells across a concentration gradient (0, 0.8, 1.6, 3.2, 6.25, 12.5, 25, 50, and 100 ppm), as shown in the Figure 3B. Within the lower concentration range of 0 to 6.25 ppm, cell viability remained at or above 100% for blank LNPs, CBD in its natural form, and CBD/LNPs, indicating minimal cytotoxic effects and a high cellular tolerance. A reduction in cell viability was observed for cells treated with the natural form of CBD at concentrations starting at 6.25 ppm, suggesting increased cytotoxicity for unencapsulated CBD.

Furthermore, CBD/LNPs maintained cell viability above 80% at concentrations up to 50 ppm. At higher concentrations (100 and 200 ppm), cell viability decreased to approximately 80% (100 ppm) or lower (200 ppm), indicating dose-dependent cytotoxicity at elevated CBD/LNP levels. Subsequent evaluations of the CBD/LNPs incorporated into hydrogels were conducted in animal models to assess biocompatibility and therapeutic efficacy.

### 2.5. Anti-Oxidant Activity of CBD/LNPs

CBD/LNPs exhibited significant anti-oxidant activity in dermal fibroblast cells subjected to oxidative stress, as shown in Figure 3C. Treatment with CBD/LNPs at concentrations of 6.25, 12.5, 25, 50, and 100 ppm resulted in a notable enhancement of antioxidant activity compared to the blank control (*p*  <  0.001 for CBD/LNP at 6.25 and 12.5 ppm; *p*  <  0.01 for CBD/LNP at 25 and 50 ppm) which refers to untreated cells exposed only to the medium without any treatment. The effect of CBD/LNPs on ROS levels was observed to be dose-dependent. Specifically, at the highest concentration of 100 ppm, ROS levels were reduced by 65% compared to the H_2_O_2_-treated control group (*p*  <  0.05). These findings indicate that CBD/LNPs possess substantial antioxidant properties, effectively mitigating oxidative stress in dermal fibroblast cells. This suggests their potential therapeutic applications in treating skin-related conditions associated with oxidative stress.

### 2.6. In Vitro Wound Healing of CBD/LNPs

**Figure 4:**
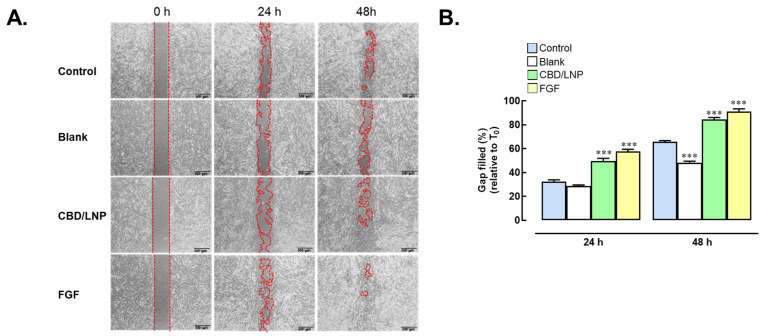
Effects of cannabidiol-loaded lipid nanoparticles (CBD/LNPs) on wound healing in fibroblast-cultured cells. (**A**) Human dermal fibroblast cells were treated with 50 ppm CBD/LNPs or 100 ng/mL FGF (positive control). A scratch wound assay was monitored at 0, 24, and 48 h post-scratch and compared to untreated control cells. (**B**) Percentage of wound gap closure at 24 and 48 h, calculated relative to the initial scratch width (T_0_). Data are expressed as mean  ±  SEM (*n*  =  3). *** *p*  <  0.001 compared to the control group.

The impact of CBD/LNPs on wound healing was investigated in dermal fibroblast cells at a concentration of 50 µg/mL, with a focus on cell migration measured through scratch wound assays (Figure 4A,B). In the non-treated control group, cell migration was recorded at 32.4 ± 3.5% after 24 h, with a notable increase to 65.7 ± 2.6% by the 48 h mark. The group treated with blank nanoparticles displayed a lower migration rate, demonstrating 28.3 ± 1.5% at 24 h and 48.0 ± 2.3% at 48 h, which were significantly lower than the control (*p* < 0.001), indicating limited efficacy in promoting cellular recovery. In contrast, the CBD/LNPs’ treatment significantly enhanced cell migration, yielding 49.0 ± 4.7% at 24 h (*p* < 0.001) and an impressive 84.5 ± 3.0% at 48 h (*p* < 0.001), compared to control, suggesting a robust stimulatory effect on fibroblast activity. The positive control group treated with FGF exhibited the highest proliferation rates, measuring 57.9 ± 3.4% at 24 h (*p* < 0.001) and 91.4 ± 3.5% at 48 h (*p* < 0.001), compared to control, underscoring the effectiveness of FGF in promoting fibroblast migration. These results indicate that CBD/LNPs significantly enhance cell migration in dermal fibroblast cells compared to both blank nanoparticles and the non-treated control. This study suggests that CBD/LNPs may serve as a promising therapeutic strategy for promoting wound healing through enhanced fibroblast activity.

### 2.7. The Effect of CBD/LNP-PVA/SA Hydrogel Scaffold on Wound Healing in Rat Skin

**Figure 5:**
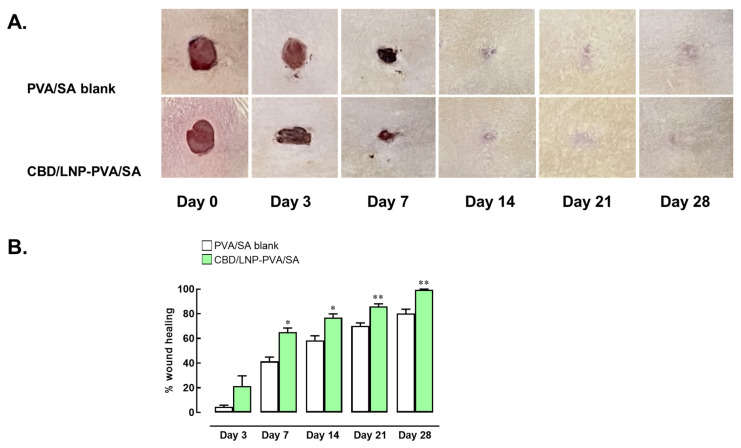
Effects of cannabidiol-loaded lipid nanoparticles incorporated in polyvinyl alcohol and sodium alginate (CBD/LNP-PVA/SA) hydrogel scaffold on wound healing in rat skin. (**A**) Time-dependent progression of wound closure in rats treated with PVA/SA hydrogel (Blank) and CBD/LNP-PVA/SA hydrogel. (**B**) Percentage of wound area contraction calculated on days 3, 7, 14, 21, and 28 post-treatment. Data are expressed as mean  ±  SEM (*n* = 3 per time point). * *p*  <  0.05 and ** *p*  <  0.01 compared to the blank group on the respective day.

On day 3, the group treated with CBD/LNP-PVA/SA hydrogel scaffold exhibited a higher percentage of wound healing than the PVA/SA hydrogel scaffold blank group, though this difference was not statistically significant. By day 7, a notable improvement in wound healing was observed in the CBD/LNP-PVA/SA hydrogel scaffold-treated group, with significantly higher healing compared to the PVA/SA hydrogel scaffold blank group (*p* < 0.05). This trend continued on day 14, as the CBD/LNP-PVA/SA hydrogel scaffold-treated group showed a statistically significant increase in wound healing relative to the PVA/SA hydrogel scaffold blank group. By day 21, the difference between the groups became even more pronounced, with the CBD/LNP-PVA/SA hydrogel scaffold treatment resulting in a marked acceleration in wound closure (*p* < 0.01). By the final assessment on day 28, wounds in the CBD/LNP-PVA/SA hydrogel scaffold-treated group were nearly completely healed, reaching approximately 100% healing, which was significantly greater than that of the PVA/SA blank group (Figure 5). These findings suggest that CBD/LNP-PVA/SA hydrogel scaffold significantly enhance wound healing over time compared to the PVA/SA hydrogel scaffold blank control, with observable improvements beginning on day 7 and near-complete healing achieved by day 28.

### 2.8. The Effect of CBD/LNP-PVA/SA Hydrogel Scaffold on Rat Skin Histopathomorphology

**Figure 6:**
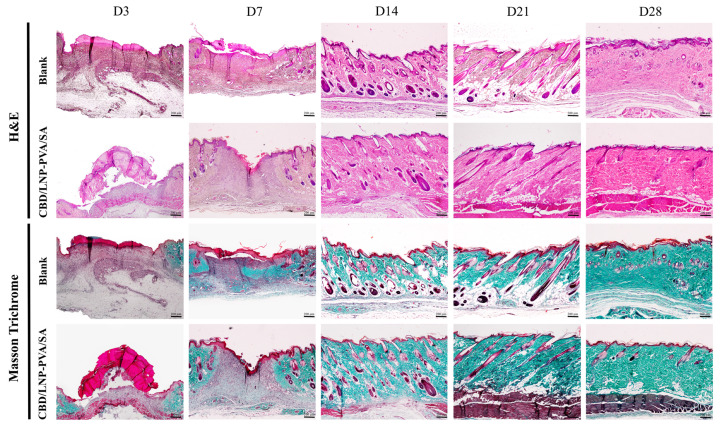
Effects of cannabidiol-loaded lipid nanoparticles incorporated into polyvinyl alcohol and sodium alginate (CBD/LNP-PVA/SA) hydrogel scaffold on histopathological changes in rat skin on days 3, 7, 14, 21, and 28 post-wounding, as assessed by H&E and Masson’s trichrome staining. Photographs show wound sections from rats treated with PVA/SA (Blank) and CBD/LNP-PVA/SA hydrogel scaffold. Hematoxylin-stained nucleic acids and nuclei, eosin-stained proteins, and collagen are visualized in blue–green. Scale bar = 200 μm.

As shown in Figure 6 and Table 2, the histological analysis demonstrated significant improvements in the wound healing parameters in the CBD/LNP-PVA/SA hydrogel scaffold treated group compared to the PVA/SA blank group at various time points. On day 3, the treatment group exhibited a significantly greater epidermal thickness and dermal thickness compared to the PVA/SA blank group (*p* < 0.001). Additionally, the number of vessels in the wound bed was slightly increased in the CBD/LNP-PVA/SA hydrogel scaffold-treated group compared to the PVA/SA blank control, while the number of inflammatory cells was significantly reduced (*p* < 0.05). By day 7, the dermal thickness remained significantly higher in the CBD/LNP-PVA/SA hydrogel scaffold-treated group compared to the PVA/SA blank group (*p* < 0.001). The collagen density also increased significantly in the treatment group compared to the blank (*p* < 0.05), along with a notable increase in vessel numbers (*p* < 0.01). Furthermore, inflammatory cell counts were significantly lower in the CBD/LNP-PVA/SA hydrogel scaffold-treated group than in the PVA/SA blank group (*p* < 0.01). Additionally, on day 14, while the epidermal and dermal thickness showed no significant differences, the collagen density in the treatment group was markedly higher than in the blank group (*p* < 0.01). The number of vessels also increased significantly in the treatment group compared to the PVA/SA blank control (*p* < 0.05), accompanied by a reduction in inflammatory cell counts. By day 21, the dermal thickness remained significantly higher in the treatment group than in the blank group (*p* < 0.001), and the collagen density was also significantly enhanced (*p* < 0.01). Inflammatory cells were further reduced in the CBD/LNP-PVA/SA hydrogel scaffold-treated group compared to the PVA/SA blank group. Finally, by day 28, there were no significant differences in the epidermal thickness, dermal thickness, collagen density, or vessel numbers between the groups, though inflammatory cells were absent in the treatment group, compared to trace levels in the blank group. These findings highlight the efficacy of CBD/LNP-PVA/SA hydrogel scaffold in promoting dermal remodeling, enhancing angiogenesis, and reducing inflammation during the early and intermediate stages of wound healing.

Therefore, the CBD/LNP-PVA/SA hydrogel scaffold effectively enhanced dermal remodeling, collagen deposition, and vascularization while reducing inflammatory responses, indicating this hydrogel’s significant role in facilitating the wound-healing process. The findings suggest that this nanoformulation may be a promising therapeutic approach for improving wound healing outcomes, particularly in conditions characterized by delayed or impaired healing.

The findings presented in this study emphasize the potential of CBD/LNPs-PVA/SA hydrogel scaffold as a novel therapeutic strategy for enhancing wound healing and addressing skin-related conditions. The favorable stability, low cytotoxicity, significant antioxidant activity, and enhanced fibroblast proliferation of CBD/LNPs contribute to the promising profile of PVA/SA or blank hydrogel scaffold for clinical applications.

As the results demonstrate, the swelling behavior of PVA/SA hydrogel scaffold is closely tied to the concentration of sodium alginate, with higher concentrations leading to enhanced water absorption [19]. The remarkable swelling ratios observed for formulations with a high sodium alginate content highlight the hydrophilic properties of alginate (Figure 1), which can significantly influence drug release kinetics and the overall therapeutic efficacy [20]. In wound healing, the ability of hydrogels to absorb exudate while maintaining moisture at the wound site is essential [21]. The swelling characteristics suggest that hydrogels containing higher concentrations of sodium alginate could provide an optimal environment for wound healing, promoting both drug delivery and moisture retention [22,23]. Subsequently, the release profiles of PVA/SA hydrogel scaffold demonstrate that the sodium alginate concentration significantly influences the release kinetics of CBD in LNPs (Figure 2). The formulation with 75% sodium alginate showed the highest release rate, which may be attributed to enhanced porosity and hydrophilicity provided by alginate [24]. The relationship between the release kinetics and alginate concentration is crucial for controlled drug delivery systems, ensuring that therapeutic agents are released in a sustained manner [17]. By optimizing the composition of PVA/SA hydrogel scaffold, the therapeutic efficacy of CBD can be maximized, supporting its use in managing wound healing.

Meanwhile, the characterization of CBD/LNPs showed that they maintain structural integrity and stability over a 2-month period at various temperatures (Table 1). The minimal variation in average particle sizes, PDI, and zeta potential values across different temperatures suggests that CBD/LNPs exhibit favorable characteristics for potential therapeutic applications. The zeta potential values, remaining around -30 mV, indicate strong electrostatic repulsion, which is essential for maintaining nanoparticle stability and preventing aggregation [15,25]. The slight increase in particle size after 2 months, although statistically insignificant, could be attributed to minor changes in the physical state of the formulation, yet this does not compromise the efficacy of the nanoparticles. The results also hint at a potential decrease in the encapsulation efficiency, which should be further investigated. Ensuring that CBD maintains its bioactive properties within the lipid nanoparticle matrix is crucial for its therapeutic efficacy [26]. Overall, the findings support the potential use of CBD/LNPs in hydrogel scaffold for sustained delivery, thereby enhancing the pharmacological effects of CBD while ensuring stability over time.

The results from the cytotoxicity evaluation reveal that CBD/LNPs exhibit a significantly lower cytotoxic profile compared to unencapsulated CBD, especially at higher concentrations (Figure 3B). The finding that cell viability remained above 80% at all concentrations of CBD/LNPs signifies the biocompatibility of these nanoparticles, making them suitable for therapeutic applications [15,27]. In contrast, the natural form of CBD showed noticeably increasing cytotoxicity at concentrations above 6.25 ppm, indicating that encapsulation within lipid nanoparticles not only protects the bioactive compound, but also enhances cellular tolerance. This biocompatibility is critical when considering therapeutic formulations for skin applications [28], where dermal fibroblasts play a vital role in wound healing and tissue regeneration [29]. The results suggest that CBD/LNPs can provide therapeutic benefits without compromising cellular health, thereby reinforcing their potential in combination with material dressings for applications in dermatology.

Furthermore, the significant antioxidant activity demonstrated by CBD/LNPs under oxidative stress conditions is a promising finding (Figure 3C). The dose-dependent reduction of ROS indicates that CBD/LNPs not only combat oxidative stress, but also have the potential to protect dermal fibroblast cells from oxidative damage. Oxidative stress is known to impede wound healing and contribute to various skin disorders, making the antioxidant properties of CBD/LNPs particularly relevant for therapeutic strategies targeting these conditions [30,31]. The ability of CBD in LNPs to reduce ROS levels by up to 65% suggests a robust mechanism for mitigating oxidative damage, which may enhance the overall skin health and facilitate faster wound healing [32]. This finding lays the groundwork for further investigation into the therapeutic role of CBD/LNPs, particularly in managing oxidative-stress-related skin conditions.

This study evaluated the impact of CBD/LNPs on fibroblast migration using the scratch wound assay, with gap closure serving as an indicator of the wound healing potential. As reported by Gurgul et al. (2024), CBD exhibits toxicity to human dermal fibroblast cells at low doses (0.75 µM) after 24 h, but promotes cell proliferation at earlier time points (6–12 h) [33]. In our study, CBD/LNP treatment did not induce a significant proliferation effect compared to the control group. The observed wound closure appears to be primarily due to enhanced cell migration, rather than an increase in cell proliferation. The substantial increase in cell migration observed in CBD/LNP-treated groups compared to controls demonstrates the ability to stimulate fibroblast activity. As shown in Figure 4, CBD/LNPs outperformed blank nanoparticles and exhibited effects comparable to positive controls, such as FGF, a well-established promoter of cell migration and proliferation [34]. The enhanced wound closure observed in the in vitro assay is primarily attributed to increased fibroblast migration promoted by CBD/LNPs, as no significant proliferation was detected in treated cells compared to the controls. These findings suggest that CBD/LNPs stimulate fibroblast migration, a critical process in wound healing, without notably affecting cell proliferation. This effect is significant, as fibroblasts play a central role in wound repair through collagen synthesis and extracellular matrix formation [29,35].

The enhanced wound closure observed in the in vitro assay is mainly attributed to the increased cell migration promoted by CBD/LNPs, as no significant proliferation was observed in the treated cells compared to the controls. This result suggests that CBD/LNPs enhance fibroblast migration, a key process in wound healing, without significantly affecting cell proliferation. This stimulatory effect on fibroblast migration and proliferation is crucial, as these cells are integral to the wound healing process, involved in collagen synthesis and extracellular matrix formation [29,35].

Keratinocytes are widely recognized as a suitable cell type for cytotoxicity assays and scrape-wound healing models due to sheet-like migration, which closely approximates wound closure in vitro [36]. In contrast, fibroblasts exhibit a distinct migratory behavior that is less conducive to mimicking re-epithelialization. Despite this, the present study emphasizes fibroblasts, given the pivotal role in extracellular matrix remodeling and cellular migration, processes critical to the early stages of wound healing [37]. Although keratinocyte migration is indispensable for re-epithelialization and overall wound closure [38], its inclusion was beyond the scope of this investigation. Future studies should address keratinocyte behavior to provide a more holistic understanding of the scaffold’s role in facilitating both early and late phases of wound healing. Based on our results, the promotion of fibroblast activity by CBD delivered via LNPs may enhance tissue regeneration and improve the healing process in skin injuries, demonstrating its potential as a therapeutic option in wound care [8,39].

In addition to validating the efficacy and safety of CBD/LNPs and the behavioral properties of the hydrogel, the in vivo results, indicating improved wound healing in rats treated with CBD/LNP-PVA/SA hydrogel scaffold, substantiate the in vitro findings (Figure 5). The statistically significant differences observed by day 7 and the marked improvement by day 28 suggest that CBD/LNPs enhance the healing process over time. These findings are consistent with previous studies showing the roles of CBDs in promoting wound healing [5]. The accelerated healing observed in the CBD/LNP-treated group likely results from a combination of enhanced fibroblast activity, antioxidant effects, and the sustained release characteristics of the hydrogels, creating a supportive environment for skin regeneration [40,41].

In histological assessments (Figure 6 and Table 2), CBD/LNP-PVA/SA hydrogel scaffold facilitate significant improvements in wound healing. The results demonstrate the therapeutic potential of CBD/LNP-PVA/SA hydrogel scaffold in promoting wound healing by improving key parameters including the dermal thickness, collagen density, and angiogenesis, while significantly reducing inflammation, particularly during the early and intermediate stages of healing. The most notable effects were observed on days 3, 7, and 14, where the treatment group consistently outperformed the blank group. By day 28, most parameters between the two groups converged, suggesting that the treatment primarily accelerates the healing process during the critical initial phases. These morphological changes highlight the therapeutic benefits of CBD in wound healing and provide insights into the mechanisms through which CBD/LNPs exert beneficial effects, such as modulating inflammatory responses, promoting fibroblast proliferation, and enhancing antioxidant activity [42]. The physiological mechanisms by which CBD/LNPs enhance wound healing appear to be multifaceted, involving cellular, biochemical, and tissue-level interactions [20]. CBD/LNPs provide a sustained release of CBD, ensuring prolonged availability at the wound site, which aids in the continuous modulation of healing processes [26].

The LNPs stabilize the CBD hydrogel scaffold, which may help maintain therapeutic concentrations of CBD, which have been reported to regulate the activity of cytokines such as IL-6 and TNF-alpha, as observed in previous studies [26,43,44]. However, this mechanism was not directly evaluated in the current study and remains a topic for future investigation. Moreover, CBD has been observed to stimulate fibroblast activity and collagen synthesis, essential steps in re-epithelialization and tissue remodeling [20]. Encapsulation within LNPs allows CBD to target and engage fibroblasts over time, promoting a steady increase in collagen deposition and extracellular matrix formation, which are critical for wound closure and tensile strength [20,45].

These results are promising for the development of CBD/LNP-based hydrogels in clinical settings, particularly in enhancing skin repair and regeneration. The insights gained from this study may pave the way for future investigations into the underlying molecular mechanisms, skin irritation, and the efficacy of CBD/NLPs across various therapeutic areas, especially in dermatology and regenerative medicine.

However, a limitation of the present study was the use of only male rats to avoid hormonal changes that could affect skin regeneration and vascular responses [46]. This approach restricts the ability to evaluate potential sex differences in wound healing and responses to CBD/LNP treatment. To provide a more comprehensive understanding of sex-based variations and ensure the broader applicability of the findings, future studies should include both male and female animals. Furthermore, the exact concentration of CBD delivered was not measured, which limits the ability to precisely attribute the observed anti-inflammatory effects. Cytokine analysis in the rat model using methods such as ELISA was not conducted in this study. Incorporating such analyses in future research could provide valuable insights into the inflammatory responses and the mechanisms underlying the therapeutic effects of CBD/LNP treatment. Additionally, further in vivo comparisons of the LNP-PVA/SA hydrogel scaffold (without CBD) are necessary to assess the feasibility of a transdermal delivery system for CBD. Such a system could potentially enhance and support wound healing synergistically with CBD’s therapeutic effects.

## 3. Conclusions

In this study we successfully incorporated CBD in a hydrogel scaffold for a wound-healing approach. A PVA/SA hydrogel scaffold exhibited a concentration-dependent swelling behavior, crucial for the controlled release of CBD, with biphasic release profiles. In addition, CBD/LNPs remained uniform and maintained stability over two months, with only minor changes in their size and zeta potential. Compared to unencapsulated CBD, CBD/LNPs showed enhanced biocompatibility by demonstrating significantly reduced cytotoxicity on dermal fibroblasts and notable antioxidant activity by effective ROS reduction. In vitro gap-closure assays indicated that fibroblasts treated with CBD/LNPs showed enhanced cell migration and proliferation, promoting wound closure. In vivo studies revealed that CBD/LNP-PVA/SA hydrogel scaffold significantly accelerated wound healing, with an improved dermal architecture, collagen deposition, enhanced angiogenesis, and reduced inflammation compared to control groups. These findings support the potential of CBD/LNP-PVA/SA hydrogel scaffold as an effective treatment for chronic wounds, warranting further investigation to optimize formulations and assess long-term efficacy in clinical settings.

## 4. Materials and Methods

### 4.1. Preparation and Physical Stability of Cannabidiol-Loaded Lipid Nanoparticles (CBD/LNPs)

CBD/LNPs were produced through a solvent injection technique. In this method, lipid nanoparticles were created by combining solvent phase and aqueous phases via microfluidization. CBD powder (sourced from Amara Asia Co., Ltd., Bangkok, Thailand) was dissolved in ethanol alongside lipid components including phosphatidylcholine and cholesterol (Lipoid GmbH, Ludwigshafen, Germany). The solvent phase was then mixed with the aqueous phase (deionized water) using a high-speed homogenizer (IKA, Staufen, Germany). The particle size was further reduced using a microfluidizer (M-110P Microfluidizer, Microfluidics Inc., Westwood, MA, USA). Mechanical force facilitated the formation of lipid nanoparticles. Ethanol was subsequently eliminated through rotary evaporation under a reduced pressure, yielding a final CBD concentration of 3 mg/mL. The stability of CBD-LNPs was assessed based on their average particle size, zeta potential, and polydispersity index (PDI), with measurements obtained using a Malvern Instruments Zetasizer Nano ZX (Malvern Panalytical Ltd., Malvern, UK) through dynamic light scattering (DLS) after 30 days and 60 days of storage at temperatures of 4 °C, 25 °C, and 40 °C.

### 4.2. Hydrogels Scaffold Preparation

The preparation of hydrogel scaffolds and hybrid systems (nanoparticles/hydrogel) was adapted from the method described by Bahadoran et al. (2020) [16]. A 10% (*w*/*v*) solution of PVA (Sigma Aldrich, St. Louis, MO, USA) was prepared by dissolving PVA in distilled water under magnetic stirring at 80 °C, followed by cooling to room temperature. Additionally, a 1.5% (*w*/*v*) SA (Sigma Aldrich, St. Louis, MO, USA) solution was made in distilled water at 40 °C. Blends of PVA and SA solutions were then created in various ratios (SA = 0, 25, 50, 75, and 100 *v*/*v* in SA/PVA mixtures) and mixed with a magnetic stirrer for 30 min, then CBD/LNPs were added at 100 ppm CBD in the mixture. The uniform gel mixtures were poured into Petri dishes, then subjected to three freeze–thaw cycles (frozen at −20 °C for 20 h, thawed at 25 °C for 4 h each cycle). The scaffolds were then immersed in 2% calcium chloride (CaCl_2_, Sigma Aldrich, St. Louis, MO, USA) for 1 h, rinsed with distilled water, and freeze-dried for 24 h to remove water and increase porosity. Finally, the scaffolds were cut into rectangular shapes for further studies.

### 4.3. Swelling Behavior and In Vitro CBD/LNP Release

To assess the water absorption capacity of the hydrogel scaffolds, their initial weights were measured immediately following freeze-drying. The scaffolds were subsequently immersed in distilled water at room temperature. At designated time intervals, the wet weights were measured after gently blotting the sample surfaces with filter paper to remove any excess water. The degree of swelling was then calculated by using the following equation.

$$Degree\;of\;swelling=\frac{\mathrm{Wet weight}-\mathrm{Dry weight}}{\mathrm{Dry weight}}\times 100$$

To assess the release of CBD nanoparticles from each hydrogel formulation, CBD/LNP was incorporated into the hydrogels at a concentration of 100 ppm CBD. The in vitro release study utilized fluorescence staining (Dil) to label the CBD/LNPs. Hydrogel scaffolds were placed in the upper section of 24 mm transwells (Corning, Berlin, Germany), with the lower section filled with PBS (pH 7.4), and incubated at 50 rpm at 37 °C. This setup allowed CBD/LNPs to diffuse into the PBS solution in the bottom chamber. At specified intervals, the amount of CBD/LNPs released into the PBS was quantified by measuring the fluorescence intensity using a SpectraMax M2 microplate reader (Molecular Devices, San Jose, CA, USA). After each measurement, fresh buffer solution was added and the cumulative release was recorded.

### 4.4. Cytotoxicity Assay

Human foreskin fibroblast cells (ATCC SCRC-1041) were cultured in DMEM (Thermo Fisher Scientific, Waltham, MA, USA) supplemented with 10% fetal bovine serum (FBS) and 100 µg/mL streptomycin (Pen&Strep). Cells were maintained at 37 °C in a 5% CO_2_ incubator. Cytotoxicity was assessed using the MTT (3-(4,5-dimethylthiazol-2-yl)-2,5-diphenyltetrazolium bromide) reduction assay. Cells were seeded at a density of 1 × 10⁴ cells per well in 96-well plates and allowed to reach 70–80% confluency before being treated with CBD/LNPs, blank/LNPs, and isolated CBD. After 24 h of incubation, 100 µL of MTT solution (1 mg/mL in PBS) was added to each well, and plates were incubated for an additional 4 h at 37 °C. The MTT solution was then removed, and the resulting formazan crystals were dissolved by adding 100 µL of DMSO to each well. Absorbance was measured at 570 nm using a SpectraMax M2 microplate reader (Molecular Devices LLC, San Jose, CA, USA).

### 4.5. Anti-Oxidation Activity

The intracellular reactive oxygen species (ROS) levels in fibroblast cells were quantified using the dichlorofluorescein (DCF) assay. In this study, fibroblasts were seeded at a density of 1 × 10⁴ cells per well in 96-well plates and cultured to 70–80% confluence prior to treatment. Cells were then treated with CBD/LNPs and blank/LNPs for 24 h. Following treatment, the medium was removed, and the cells were incubated with 50 µg/mL H_2_O_2_ in fresh medium for 30 min and washed twice with PBS to induce ROS generation. Cells were then incubated with 25 µM DCFH_2_-DA in a medium for an additional 30 min to allow ROS detection. After a final wash with PBS, DCF fluorescence was measured using a SpectraMax M2 microplate reader (Molecular Devices LLC, San Jose, CA, USA) to quantify the ROS levels.

### 4.6. Wound Healing Assay

The in vitro wound healing assay was conducted by evaluating fibroblast cell migration across gap surfaces. Fibroblast cells were seeded into 4-well micro-dishes with inserts (ibidi GmbH, Gräfelfing, Germany) containing DMEM medium, allowing them to expand nearly confluent monolayers. Following this, the silicon gaskets were removed to create a consistent gap in the cell monolayer. For each experimental condition, MEM medium (control group), blank lipid nanoparticles (without CBD), fibroblast growth factor (FGF, 100 ng/mL, positive control), and CBD/LNP (50 ppm) were added to separate dishes (3 dishes per conditions), which were then incubated at 37 °C with 5% CO_2_ for 24 and 48 h. Cells were subsequently fixed with 4% paraformaldehyde for 15 min and washed three times with cold PBS. To assess cell migration, five representative images of the gap area were taken per dish, and the extent of migration was quantified using ImageJ software (version 1.54 k, National Institutes of Health, 2024).

### 4.7. Animals

In accordance with the guidelines set by the Association for the Assessment and Accreditation of Laboratory Animal Care (AAALAC) International, this study adhered to the 3Rs principle—Replace, Reduce, and Refine—by minimizing both the number of animals used and their discomfort. A total of 15 adult male Wistar rats, each weighing between 190 and 210 g and aged 8 weeks, were selected based on previous studies to determine the appropriate sample size [47,48]. Rats were randomly assigned to groups corresponding to treatment time points on days 3, 7, 14, 21, and 28. The rats were sourced from Nomura Siam International Co., Ltd., Bangkok, Thailand, and were housed in groups of 2–3 per cage. Rats were kept in a controlled laboratory environment under a 12 h light/12 h dark cycle, with a temperature maintained at 24 ± 1 °C and relative humidity at 54–55%. Rats had ad libitum access to standard rodent chow (CP Co., Ltd., Bangkok, Thailand) and sterile reverse osmosis water. All experimental procedures in the animal study were approved by the Animal Care Committee at Thammasat University, Thailand, under ethical clearance certified by the International Laboratory Accreditation Cooperation (protocol number: 01/2023).

### 4.8. Surgical Procedures and Treatments

After a 7-day acclimation period, each rat underwent surgery to create two full-thickness skin wounds, each measuring 1 cm in diameter, on the dorsal surface. The upper wound was designated as the control, while the lower wound received treatment with CBD/LNP-PVA/SA hydrogel scaffold. The procedure was conducted under isoflurane anesthesia, with strict aseptic protocols observed throughout [47,48]. Signs of infection were monitored, and tramadol injections were administered daily for the first three days post-surgery to manage pain. Rats displaying weight loss, a reduced food or water intake, or severe infection were to be excluded from the study, although no such issues were encountered.

Each rat had two wounds, one treated with CBD/LNP-PVA/SA hydrogel scaffold (1 × 1 cm) and the other with PVA/SA hydrogels as a blank control, serving as an internal control. Wounds were cleaned daily with normal saline, and treatments were applied directly to the wound and the surrounding area. On days 3, 7, 14, 21, and 28, the rats were anesthetized with an overdose of isoflurane for photographing and tissue collection. Wound size changes, calculated as percentages relative to the baseline ([baseline wound size-measured wound size]/baseline wound size) × 100], were used to evaluate the healing progress. Skin samples were collected at each time point for a histopathological analysis to assess the effects of CBD/LNP-PVA/SA hydrogel scaffold treatment on wound healing.

### 4.9. Histological Analysis

Histopathological images stained with H&E and Masson’s Trichrome were obtained from formalin-fixed, paraffin-embedded skin tissue blocks to evaluate the inflammatory responses, epidermal thickness, and collagen deposition, particularly along the epidermal boundary. The H&E-stained images, imported in a JPG format, were analyzed using specialized software to quantify the vessel and inflammatory cell counts per field of view (FOV), with counts conducted across 10 FOVs per group at 400× magnification. The skin thickness was measured at 100× magnification (scale bar = 200 μm) by manually drawing line segments. Masson’s Trichrome staining was used to visualize collagen deposition, indicated by green coloration, to assess the collagen distribution and density [47,48].

### 4.10. Statistical Analysis

Data are presented as means ± SEM. Differences between groups were analyzed using an unpaired t-test and ANOVA with Dunnett’s post hoc test, with significance set at *p* < 0.05. All statistical analyses and graph plotting were performed using GraphPad Prism 10.0 (GraphPad Software Inc., San Diego, CA, USA).

## Figures and Tables

**Table 1:** The stability of CBD/LNPs at storage temperatures.

CBD/LNPs	1 Month			2 Months		
	Size (nm)	PDI	Zeta Potential (mV)	Size (nm)	PDI	Zeta Potential (mV)
Temperature (°C)						
4	131.90	0.161	−34.80	135.70	0.182	−32.00
25	136.80	0.173	−31.80	136.70	0.192	−30.90
40	132.50	0158	−31.40	134.10	0158	−27.30

The values, including size, polydispersity index (PDI), and zeta potential of CBD/LNPs, were measured to assess stability under different conditions when stored at various temperatures (4 °C, 25 °C, and 40 °C) over one and two months.

**Table 2:** Histological wound healing parameters in rats treated with CBD/LNP-PVA/SA hydrogels.

Time/Group	Parameters				
	Epidermal Thickness (µm)	Dermal Thickness (µm)	Collagen Density (%)	Number of Vessels in Wound Bed (per Field)	Inflammatory Cell (per Field)
Day 3					
PVA/SA blank	229.94 ± 23.14	778.98 ± 27.69	8.86 ± 4.25	17.67 ± 2.67	10.00 ± 1.15
CBD/LNP-PVA/SA	75.72 ± 30.66 ***	1044.74 ± 19.45 ***	8.98 ± 2.52	22.00 ± 1.53	6.00 ± 0.58 *
Day 7					
PVA/SA blank	96.95 ± 9.78	862.98 ± 10.59	10.05 ± 1.36	23.00 ± 1.15	7.33 ± 0.58
CBD/LNP-PVA/SA	90.70 ± 3.68	1055.46 ± 13.13 ***	17.08 ± 1.37 *	34.67 ± 1.45 **	3.00 ± 0.58 **
Day 14					
PVA/SA blank	45.51 ± 1.65	724.67 ± 7.56	15.11 ± 0.69	11.00 ± 0.58	3.00 ± 0.58
CBD/LNP-PVA/SA	41.38 ± 1.21	713.65 ± 7.05	25.91 ± 1.33 **	17.00 ± 1.53 *	1.00 ± 0.58
Day 21					
PVA/SA blank	48.64 ± 2.02	777.21 ± 16.57	19.69 ± 0.72	5.33 ± 0.33	1.00 ± 0.58
CBD/LNP-PVA/SA	44.38 ± 1.03	1068.88 ± 13.17 ***	31.09 ± 1.87 **	6.00 ± 0.58	0.33 ± 0.33
Day 28					
PVA/SA blank	40.36 ± 1.72	600.39 ± 8.63	31.87 ± 1.47	5.00 ± 0.58	0.33 ± 0.33
CBD/LNP-PVA/SA	42.95 ± 1.79	574.45 ± 5.97	32.16 ± 1.59	5.67 ± 0.33	0.00 ± 0.00

Data are expressed as mean  ±  SEM (*n* = 3 per time point). Quantitative analysis was performed by counting features within 10 high-power fields (HPF) at 400× magnification. * *p*  <  0.05, ** *p*  <  0.01 and *** *p*  <  0.001 compared to the PVA/SA blank control group on the respective day.

## Data Availability

The original contributions presented in this study are included in the article. Further inquiries can be directed to the corresponding author.
